# Leisure time physical exercise during pregnancy and the risk of miscarriage: a study within the Danish National Birth Cohort

**DOI:** 10.1111/j.1471-0528.2007.01496.x

**Published:** 2007-09-18

**Authors:** M Madsen, T Jørgensen, ML Jensen, M Juhl, J Olsen, PK Andersen, A-M Nybo Andersen

**Affiliations:** a Department of Child Health, National Institute of Public Health Copenhagen K, Denmark; b Department of Epidemiology, School of Public Health, UCLA Los Angeles, CA, USA; c Department of Biostatistics, University of Copenhagen Copenhagen K, Denmark

**Keywords:** Cohort study, fetal death, physical exercise, pregnancy

## Abstract

**Objective:**

To examine the association between leisure time physical exercise during pregnancy and the risk of miscarriage.

**Design:**

Prospective study with elements of retrospective data collection.

**Setting:**

Denmark 1996–2002.

**Population:**

A total of 92 671 pregnant women enrolled in the Danish National Birth Cohort and interviewed subsequently.

**Methods:**

Data on exercise during pregnancy and potential confounders were obtained through computer-assisted telephone interviews either during pregnancy or after an early miscarriage. Outcome of pregnancy was identified by register linkage. Using Cox regression analysis, we estimated the hazard ratio (HR) of miscarriage according to weekly amount of exercise and the type of exercise. The HR was estimated for <11, 11–14, 15–18, and 19–22 weeks of gestation, respectively.

**Main outcome measures:**

Miscarriage, defined as fetal loss before 22 completed weeks of gestation.

**Results:**

A stepwise increasing relation was found between amount of exercise and risk of miscarriage, where risk of miscarriage increased by amount of exercise up to HR = 3.7 (95% CI 2.9–4.7) for women who exercised more than 7 hours per week compared with nonexercisers. Particularly ‘high-impact exercise’ was associated with an increased risk of miscarriage. No association was seen between exercise and risk of miscarriage after 18 weeks of gestation.

**Conclusions:**

This study suggests that exercise early in pregnancy is associated with an increased risk of miscarriage. The results should, however, be interpreted cautiously as potential bias arising from retrospective data collection may explain part of the association.

*Please cite this paper as:* Madsen M, Jørgensen T, Jensen M, Juhl M, Olsen J, Andersen P, Nybo Andersen A. Leisure time physical exercise during pregnancy and the risk of miscarriage: a study within the Danish National Birth Cohort. BJOG 2007;114:1419–1426.

## Introduction

Physical exercise has gained increasing popularity among women in the fertile age, and as a result, many women ask for medical advice on whether or not they can continue to exercise throughout their pregnancy.^1^ Guidelines in countries such as the USA, Great Britain and Denmark are currently recommending physical activity during pregnancy at a level similar to that of the nonpregnant population. Physical exercise during pregnancy is known to have beneficial effects on numerous health outcomes, including a decreased risk of pre-eclampsia^2^,^3^ and gestational diabetes,^4^ but whether or not such effects apply to the health of the fetus remains unclear.^5^ The effect of leisure time physical activity during pregnancy should therefore be investigated to make antenatal care counselling on this subject as evidence based as possible.

Pathways which have been suggested to mediate a potential effect of maternal exercise on fetal health include: (1) reduction of placental blood flow due to redistribution of blood to the working muscles,^6^–^8^ (2) exercise-induced hyperthermia,^9^ (3) exercise-induced release of hormones stimulating uterine contractility,^10^,^11^ and (4) fetal hypoglycaemia as a result of increased glucose uptake in exercising muscles.^12^–^14^ All of these physical responses may potentially have adverse effects on pregnancy outcome. Several animal experiments have given support to these hypotheses,^6^,^7^,^9^,^14^ while human studies are less conclusive.^8^,^10^–^12^,^15^,^16^ Only few studies have specifically addressed the association between exercise during pregnancy and miscarriage. In the existing body of literature, exercise during pregnancy has generally not been associated with miscarriage,^17^–^19^ and one case–control study has even reported a protective effect of exercise during pregnancy.^20^ In contrast, Hjollund *et al.*^21^ found an increased risk of early miscarriage among women who reported a high physical strain around the time of implantation of the embryo.

Furthermore, lay people have tried to use excessive physical exercise as abortificant, and older literature mentions physical activity (e.g. jumping, running, and horseback riding) as a cause of miscarriage.^22^

Considering the relatively sparse literature and the somewhat inconsistent results, we wanted to examine the association between exercise during pregnancy and miscarriage in a large population-based cohort. The association was investigated both for the time spent on physical exercise and for the type of exercise.

Before initiating this study, permission was obtained from Denmark's National Scientific Ethics Committee and the Danish Data Protection Board.

## Methods

### Study design and population

The present study was based on data from the Danish National Birth Cohort (DNBC), which is a nationwide study of pregnant women and their offspring. Between 1996 and 2002 pregnant women were enrolled in the cohort at their first antenatal visit to the GP, where they received written information about the DNBC. The women were included in the cohort when they had signed and returned an informed consent form. A woman was considered eligible to the study if she was pregnant, wished to carry the pregnancy to term, and if her language skills enabled her to give an interview in Danish. Approximately 60% of all women received an invitation to the study, and of these, we estimate that about 60% accepted the invitation. This gives a participation rate of about 35% of all pregnancies in the period of enrolment.

During the study period, Danish women participated in the cohort with 100 422 pregnancies out of which we have data on 92 721. For the present study, 50 pregnancies were excluded since these were ectopic pregnancies or hydatidiform moles, which per definition could not result in a miscarriage. Thus, a total of 92 671 pregnancies were eligible for analysis.

Information about a number of exposures was obtained by means of computer-assisted telephone interviews. The first telephone interview, which forms the basis of this study, was scheduled to take place in gestational weeks 12–16. Women, who had already miscarried by the time of this interview, were asked to give a ‘case interview’, similar to the ordinary pregnancy interview.

Thus, the data for this study were based on a prospectively recruited cohort, however, for exposure data collection, the interview had in some of the cases to be conducted after the miscarriage (for further details on the DNBC see Olsen *et al.*).^23^

### Measurement of exposure

Self-reported information on leisure time physical exercise was based upon the following questions:

1. ‘Now that you are pregnant do you engage in any kind of exercise?’
  If a woman answered ‘yes’ she was asked:
2. ‘What kind of exercise do you engage in?’
3. ‘How many times a week do you engage in… (answer in question 2)?’
4. ‘How many minutes a time do you engage in… (answer in question 2)?’
5. ‘Do you engage in other kinds of exercise?’

A positive answer to the last question released a loop with the above questions, which continued until a negative response was given. All questionnaires are available in an English version at www.bsmb.dk.

These questions made it possible for us to obtain detailed information on several different types of exercise. The answers to the questions were combined into a measure of amount of exercise expressed by the total number of minutes of exercise per week. Amount of exercise was subsequently categorised into the following categories: 0, 1–44, 45–74, 75–149, 150–269, 270–419, and 420+ minutes/week, where the middle category was an approximation to the amount of exercise recommended in existing antenatal care guidelines for pregnant women in Denmark (30 minutes/day).^24^ For an analysis of the association between the type of exercise and miscarriage, we categorised the women according to the type of exercise most often performed. Performance of one specific type of exercise was assigned if engagement in this type exceeded 50% of a woman's total exercise engagement. The predefined categories of exercise in the questionnaire were: aerobic for pregnant women, dance, aerobic, bicycling, walking/hiking, jogging, ball games, swimming, workout/fitness training, badminton, tennis, and horseback riding. Besides this, there was an open category for other types of exercise not fitting into the *a priori* categories (e.g. rock climbing or roller skating). We divided the different types of exercise into six categories: ‘high impact’ (jogging, ball games, and racket sports), ‘low impact’ (aerobic for pregnant women, aerobic, dance, and walking/hiking), ‘workout/fitness training’, ‘bicycling/horseback riding’, ‘swimming’, and ‘nonclassifiable types of exercise’. In case a woman engaged equally in two or more types of exercise, she was classified as a ‘mixed exerciser’. Low-impact activities are activities where at least one foot is on the ground at all times, while in high-impact activities, there are moments where no parts of the body touches the ground.

### Measurement of covariates

The interview included questions on a large number of other exposures, and potential confounders were selected on the basis of their association to miscarriage in existing literature on the subject. Potential confounders were: maternal age (<20, 20 to <25, 25 to <30, 30 to <35, 35 to <40, and 40+ years), number of previous miscarriages (0, 1, 2, 3+), employment/educational status (longer higher education, mean higher education, skilled work, unskilled work, studying, unemployed, and unable to classify), coffee consumption during pregnancy (0, >0 to <2, 2 to <4, and 4+ cups/day), smoking during pregnancy (0, >0 to <10, and 10+ grams of tobacco/day), alcohol consumption during pregnancy (0, 0.5 to <1, 1 to <3, 3 to <5, and 5+ drinks/week), occupational physical strain (predominantly standing/walking or lifting more than 10 kg more than ten times/day) (no, yes), ever had a diagnosis of eating disorder (no, yes), pre-pregnant body mass index (<18.5, 18.5 to <25, 25 to <30, and 30+ kg/m^2^), fertility treatment prior to this pregnancy (no, yes), parity (0, 1+), chronic disease (no, yes), and gravidity (0, 1+).

### Measurement of outcome

The outcome measure of interest was miscarriage, defined as a nondeliberate fetal death of an intrauterine pregnancy before 22 completed weeks of pregnancy.^25^,^26^ By linking cohort data to the Civil Registration System and the Danish Medical Birth Registry, we identified all live births and stillbirths. Other pregnancy outcomes were identified through the National Discharge Registry. The National Discharge Registry keeps information on all discharge diagnoses from Danish hospitals for inpatients as well as outpatients. If these registers had no outcome for a certain pregnancy, the woman in question was contacted. This was the case for less than 1% of the pregnancies.

### Statistical analyses

Cox regression models were used to estimate the hazard ratios (HRs) of miscarriage according to exercise during pregnancy. The time variable in the model was self-reported gestational age measured in days since last menstrual period. The model allows for delayed entry, thereby taking into account the variation in gestational age of the women at the time of recruitment. Follow up ended at the time of miscarriage, other pregnancy outcomes (induced abortion or live birth), emigration, and maternal death or at 22 completed weeks of pregnancy, whatever came first. To adjust for potential biases arising from the fact that some women entered the study early in pregnancy and others later, we stratified data in the Cox regression model by pregnancy week at inclusion in the study. Because some of the women participated in the study with more than one pregnancy (*n* = 7235), we used robust standard errors to correct for dependency between observations.^27^

We estimated the HR of miscarriage according to weekly amount of exercise performed during pregnancy and according to the type of exercise most often performed, using nonexercisers as the reference. The analyses were repeated on a subcohort consisting of only prospectively interviewed women using gestational age at interview as the time of entry. The change-in-estimate method was used to assess which of the potential confounders actually did confound the analyses. Covariates were excluded one by one from a predefined model including maternal age and previous miscarriages if they did not change the HR between main exposure and miscarriage by more than 5%.^28^ In the analysis of type of exercise, we adjusted for amount of exercise. The interpretation of the risk estimates in this analysis is therefore the risk of miscarriage in women engaging in a given type of exercise for 75–269 minutes/week compared with nonexercisers. Furthermore, the HRs were estimated for four gestational subperiods: <11, 11–14, 15–18, and 19–22 weeks.

Finally, we performed two analyses on subcohorts to assess the effect of potential unknown confounding. One analysis included only primigravid women who had waited less than 12 months to become pregnant and who had no previous experience concerning their fecundity. The other analysis excluded all women with some kind of chronic or serious illness, for example hypertension or musculoskeletal disease.

All data handling and statistical analyses were performed using SAS V8.2 for Windows (SAS Institute Inc., Cary, NC, USA).

## Results

**Figure 1:**
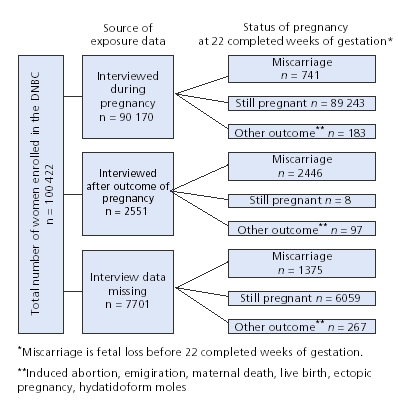
Source of interview data and status of pregnancy after 22 weeks of gestation in the study of physical exercise and risk of miscarriage among all pregnant women enrolled the DNBC (*n* = 100 422).

A total of 100 422 pregnancies were enrolled in the DNBC and 92 671 of the women participated in the first pregnancy interview. Of these interviews, 2551 were case interviews carried out after a miscarriage. Among the 92 671 pregnancies, 3187 resulted in a miscarriage (Figure 1).

Table 1 shows the frequency of weekly amount of physical exercise during pregnancy and the distribution of covariates according to this variable. Approximately 47% of all women reported that they exercised during pregnancy, and the most frequently reported amount of exercise was 75–149 minutes/week. Low-impact exercise was most frequently performed (29%) followed by bicycling/horseback riding (28%), and swimming (21%).

**Table 1:** Distribution of maternal characteristics according to amount of physical exercise during pregnancy among pregnant women in the DNBC (*n* = 92 671)

	Number (%)	Physical exercise in minutes/week						
		
		0	1–44	45–74	75–149	150–269	270–419	420+
		
		(%)	(%)	(%)	(%)	(%)	(%)	(%)
All	92 721* (100)	63.3	4.8	8.8	11.0	7.8	2.9	1.5
Maternal age (years)								
<20	948 (1.0)	1.2	0.6	0.9	0.6	0.9	0.8	1.6
20 to <25	11 109 (12.0)	12.1	10.9	12.3	11.0	11.9	13.0	15.7
25 to <30	38 347 (41.4)	39.6	46.2	45.3	44.1	44.1	43.7	39.6
30 to <35	31 454 (33.9)	34.9	32.5	31.9	33.6	31.8	30.8	29.8
35 to <40	9825 (10.6)	11.2	9.0	8.8	9.9	10.1	10.6	11.2
40+	979 (1.1)	1.1	0.9	0.8	0.8	1.3	1.3	2.1
**Previous miscarriages**								
0	74 813 (80.7)	78.9	82.9	83.4	84.0	84.6	84.7	82.7
1	13 454 (14.5)	15.6	13.5	13.2	12.7	12.1	11.5	12.8
2	3135 (3.4)	3.9	2.8	2.6	2.3	2.4	2.5	3.2
3+	1270 (1.4)	1.6	0.8	0.8	1.0	0.9	1.3	1.4
**Parity**								
0	43 567 (47.0)	41.1	54.6	53.8	57.1	59.4	63.0	63.0
1+	49 100 (53.0)	58.9	45.4	46.2	42.9	40.6	37.0	37.0
**Mode of interview**								
Prospective	90 151 (97.3)	98.0	98.1	97.5	96.0	94.9	94.1	91.7
Retrospective	2514 (2.7)	2.0	1.9	2.5	4.0	5.1	5.9	8.3
Type of exercise**								
High impact	2251 (6.6)	—	5.9	9.1	6.5	5.7	5.4	2.7
Low impact	9724 (28.6)	—	9.9	29.5	33.8	29.6	28.5	38.2
Workout/fitness training	1546 (4.5)	—	1.5	3.7	5.6	5.9	5.6	2.4
Bicycling/horseback riding	9490 (27.9)	—	12.8	12.5	25.6	38.4	39.9	31.8
Swimming	7215 (21.2)	—	64.1	34.7	12.4	3.5	1.6	0.4
Nonclassifiable types of exercise	3744 (11.0)	—	4.6	8.6	13.5	13.0	12.8	13.9

* The number of observations for each covariate may not sum to this number because of missing values.

** The distribution shown is the distribution among the 34 075 women who exercised.

**Figure 2:**
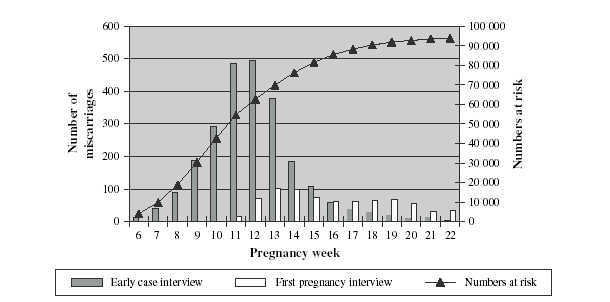
Number of pregnancies at risk according to pregnancy week, and number of miscarriages and source of interview information according to pregnancy week among women in the DNBC.

Figure 2 shows the number of pregnancies at risk according to gestational age and the number of miscarriages according to gestational week and type of interview. As expected most of the case interviews represent the earliest miscarriages (Figure 2).

Table 2 shows the association between time spent on exercise during pregnancy and the risk of miscarriage in four subperiods of gestation. The main results based on the total data material showed that an increasing amount of time spent on exercise was associated with a greater risk of miscarriage compared with nonexercisers. Exercising 1–44 minutes/week was not associated with an increased risk of miscarriage. The overall HR was 1.0 (95% CI 0.8–1.2) (results not shown). The largest risk estimate was seen in women with miscarriages in 11–14 weeks who exercised more than 419 minutes/week (HR = 3.7, 95% CI 2.9–4.7). However, no difference in the risk of miscarriage in 19–22 weeks was found between women who exercised at any amount and those who did not exercise (Table 2).

**Table 2:** HRs* of miscarriage in four gestational periods according to amount of physical exercise during pregnancy among women in the DNBC (*n* = 92 671)

Weekly amount of physical exercise (minutes)	HR			
	
	<11 weeks	11–14 weeks	15–18 weeks	19–22 weeks
	*n* = 38 489 (miscarriages = 621)	*n* = 72 638 (miscarriages = 1830)	*n* = 85 093 (miscarriages = 495)	*n* = 88 360 (miscarriages = 231)
	HR (95% CI)	HR (95% CI)	HR (95% CI)	HR (95% CI)
0	1 (ref)	1 (ref)	1 (ref)	1 (ref)
1–44	1.0 (0.7–1.6)	1.1 (0.8–1.4)	0.8 (0.5–1.3)	0.9 (0.5–1.8)
45–74	1.1 (0.8–1.5)	1.4 (1.2–1.7)	1.1 (0.8–1.5)	0.9 (0.5–1.4)
75–149	1.8 (1.4–2.3)	1.9 (1.7–2.2)	1.4 (1.1–1.9)	1.1 (0.7–1.7)
150–269	2.2 (1.7–2.8)	2.3 (2.0–2.7)	1.5 (1.1–2.1)	1.0 (0.6–1.6)
270–419	2.7 (1.9–3.7)	2.9 (2.4–3.5)	1.7 (1.1–2.7)	0.8 (0.3–2.0)
420+	3.1 (2.0–4.6)	3.7 (2.9–4.7)	2.9 (1.8–4.7)	0.6 (0.2–2.6)

* Adjusted for maternal age, previous miscarriages, and previous births.

Risk estimates based only on the prospective data material were not as large as in the total data material and hardly any statistically significant results emerged. However, there still seemed to be a slight upward trend in 11–14 weeks and to a smaller degree in 15–18 weeks (Table 3). As seen in the total data material, no significant relationship between amount of exercise and miscarriage was found after 18 weeks of gestation. Table 4 shows the association between the most frequently performed type of exercise and the risk of miscarriage in different periods of pregnancy. In the analysis based on the total data material, most types of exercise except swimming were significantly associated with an increased risk of miscarriage in the first two periods of pregnancy. Weight bearing types of exercise showed the largest HRs. Thus, high-impact exercise for 75–269 minutes/week was associated with an increased risk of miscarriage showing HRs up to 4.7 (95% CI 3.3–5.3), but low-impact exercise and workout/fitness training for 75–269 minutes/week approximately doubled the risk of miscarriage compared with nonexercisers. In addition, a moderately elevated risk was seen for bicycling/horseback riding, both nonweight bearing types of exercise.

**Table 3:** HRs* of miscarriage in three gestational periods according to amount of physical exercise during pregnancy, restricted to women with prospectively collected interview information in the DNBC (*n* = 90 170)

Weekly amount of physical exercise (minutes)	HR		
	
	11–14 weeks	15–18 weeks	19–22 weeks
	
	*n* = 23 599 (miscarriages = 286)	*n* = 55 694 (miscarriages = 263)	*n* = 76 830 (miscarriages = 186)
	
	HR (95% CI)	HR (95% CI)	HR (95% CI)
0	1 (ref)	1 (ref)	1 (ref)
1–44	0.9 (0.4–1.7)	1.1 (0.6–2.0)	1.0 (0.5–2.0)
45–74	1.4 (1.0–2.1)	1.0 (0.7–1.6)	0.7 (0.4–1.4)
75–149	1.2 (0.9–1.8)	1.1 (0.8–1.7)	1.0 (0.6–1.6)
150–269	1.1 (0.7–1.7)	1.1 (0.7–1.8)	0.7 (0.4–1.4)
270–419	1.7 (1.0–2.9)	1.2 (0.6–2.4)	0.6 (0.2–1.9)
420+	0.5 (0.1–2.0)	1.4 (0.6–3.4)	—

* Adjusted for maternal age, previous miscarriages, and previous births.

**Table 4:** HRs*,** of miscarriage in four gestational periods according to type of physical exercise during pregnancy among women in the DNBC (*n* = 92 671)

Type of preferred physical exercise	HR							
	
	<11 weeks (*n* = 38 489)		11–14 weeks (*n* = 72 638)		15–18 weeks (*n* = 85 093)		19–22 weeks (*n* = 88 360)	
	
	Cases	HR (95% CI)	Cases	HR (95% CI)	Cases	HR (95% CI)	Cases	HR (95% CI)
No exercise	319	1 (ref)	907	1 (ref)	290	1 (ref)	154	1 (ref)
High impact***	49	3.6 (2.5–5.2)	153	4.2 (3.4–5.2)	23	2.1 (1.2–3.5)	6	1.2 (0.5–3.0)
Low impact****	109	2.0 (1.4–2.6)	298	1.9 (1.6–2.3)	61	1.2 (0.8–1.8)	22	0.9 (0.5–1.8)
Workout/fitness training	20	2.1 (1.3–3.4)	50	1.9 (1.4–2.6)	16	2.0 (1.2–3.6)	8	2.3 (1.0–5.2)
Bicycling/horseback riding	79	1.3 (0.9–1.7)	281	1.7 (1.4–2.0)	67	1.3 (0.9–1.9)	16	0.7 (0.4–1.4)
Swimming	25	0.8 (0.5–1.3)	84	0.8 (0.6–1.1)	23	0.7 (0.4–1.2)	16	0.9 (0.4–1.9)
Nonclassifiable	20	1.0 (0.6–1.6)	57	0.9 (0.7–1.2)	15	0.8 (0.4–1.4)	9	1.0 (0.4–2.2)

Cases, miscarriages.

* The hazard ratios presented express the relative risk of miscarriage among women engaging in a given type of exercise for 75–269 minutes/week compared with nonexercisers.

** Adjusted for amount of exercise, maternal age, previous miscarriage, and previous births.

*** Jogging, ball games, and racket sports.

**** Aerobic, aerobic for pregnant women, dancing, and walking/hiking.

In contrast to these results, swimming for 75–269 minutes/week showed a decreased risk of miscarriage compared with nonexercisers with an overall HR of 0.8 (95% CI 0.7–1.1) (data not shown).

Generally, the HRs of miscarriage according to type of exercise seemed to decrease over gestational time, so that the HRs for most of the types of exercise equalled one in the period of 19–22 gestational weeks, except workout/fitness training. In the analysis including only prospectively collected exposure information, the estimated HRs were smaller and with considerably wider confidence limits. However, high-impact exercise for 75–269 minutes/week was still statistically significant with a HR of 1.8 (95% CI 1.0–3.6) in 11–14 weeks of gestation (data not shown). In the analyses based on subcohorts with primigravid women and women with no chronic or serious illnesses, respectively, the association between the amount of exercise and miscarriage was hardly unchanged (data not shown).

## Discussion

In this study based on data from nearly 93 000 women, a dose-response relation was seen for the association between amount of weekly exercise and the risk of miscarriage early in pregnancy. Certain types of exercise, and particularly high impact types of exercise, were found to be associated with a higher risk of miscarriage. In the analyses based only on prospectively collected exposure data, the association did, however, attenuate, indicating a certain degree of recall bias. An alternative explanation to recall bias may be that exercise only in the early stages of pregnancy has an adverse effect on pregnancy outcome. In this case, the difference in the HRs between the analyses based on the total data material and the subcohort of only prospectively collected data is not as much a result of the mode of data collection as a reflection of the fact that the total data material encompasses the very early miscarriages. Even within the subperiods of gestational age the miscarriages occur earlier for the pregnancies with retrospectively collected exposure information than for pregnancies with a first pregnancy interview (Figure 2). In addition, we did see a positive trend in the association between exercise and the risk of miscarriages in the earliest period of pregnancy (gestational weeks 11–14) in the subcohort using prospectively collected data only.

Nevertheless, retrospectively collected exposure data do involve a potential validity problem, and the data clearly showed signs of recall bias, that is the women's knowledge of their miscarriage somehow have affected the way they report on their exercise habits in the case interviews. While this mechanism may operate for soft data, such as the time spent on exercise, it is less likely that this should be the case for the type of exercise reported. Hence, it is unlikely that a woman would report jogging if she indeed engaged in swimming. Consequently, the finding of associations between certain, mainly strenuous, types of exercise and risk of miscarriage may question the notion of recall bias. Selection bias could be another explanation of the difference in the risk estimates between the two modes of data collection. This owes to the fact that only two-thirds of the women who had miscarried before the execution of the pregnancy interview agreed to give a case interview (Figure 1). Sensitivity analyses where missing data were imputated have, however, shown that an association between exercise in pregnancy and miscarriage persisted in all of the examined scenarios (results not shown).

It is difficult to investigate very early miscarriages using prospectively collected exposure information, since the time period, in which collection of exposure information must take place, that is the time from detection of pregnancy to the occurrence of an early miscarriage, is short. We consider the data at hand valuable for a number of reasons. First of all, it is a very large study population that allows us to study rare outcomes, which for practical reasons would be difficult in a clinical design. In addition, the observational design of this study makes it possible to examine pregnant women's real-life exposures, which can render some important insights not obtainable in clinical studies. Lastly, the prospective design of the study has limited the selection of women into the cohort, and the early recruitment of the women has allowed us to study early miscarriages. In conclusion, despite the mentioned potential validity problems, we do consider them to be less severe than in a traditional case–control design.

The association between exercise and risk of miscarriage need not necessarily reflect a causal mechanism. Nausea is known to be significantly less common in pregnancies that end in miscarriage,^29^ and if women with nausea are more likely to quit exercising than those who are not suffering from nausea because of a malfunctioning pregnancy, it is a potential source of bias. Information on early nausea was, however, lacking in this study.

Information on pre-pregnancy exercise habits could also have been relevant as pre-pregnancy exercise habits could be suspected to modify the effect of exercise during pregnancy. In addition, exercise habits around the time of implantation might also have been of interest. Furthermore, information on exercise intensity was lacking in the exposure measure. Intensity could be regarded as an important dimension of exercise as different intensities may release different physical responses. The examination of the various types of exercise may, however, be a rough approximation of the different levels of intensity.

Only few previous studies have investigated the association between exercise and miscarriage. The only study, which clearly supports our findings, is a cohort study, which concluded that self-reported physical strain around the time of implantation (days 6–9 after ovulation) was associated with an increased risk of miscarriage (HR 2.5, 95% CI = 1.3–4.6).^21^ In contrast, Latka *et al.*^20^ found a reduced risk of miscarriage with no chromosome defect in women who exercised compared with those who did not (OR = 0.5, 95% CI = 0.3–1.0). The case–control design was, however, based on a hypothesis that exercise cannot lead to chromosome aberrations in the fetus, as the control group consisted of women with miscarriages with chromosome aberrations. This assumption may be questioned since mode of action is unknown. In a small prospective study, Clapp^19^ found no statistically significant difference in risk of miscarriage between recreational runners (*n* = 49), aerobic dancers (*n* = 39) and a control group of active women, who had stopped exercising before the time of conception (*n* = 29). The study population was in excellent condition and had been exercising for years prior to the pregnancy, and the results may not be representative of the population at large. Two other studies have only investigated late miscarriages.^17^,^18^

Despite potential validity problems due to retrospective data collection, the results of this study suggest that leisure time exercise during pregnancy, and particularly high-impact exercise, is associated with an increased risk of miscarriage in the early stage of pregnancy, while exercise in later periods of gestation does not affect the risk of miscarriage. The mode of action is unknown, but the fact that high-impact exercise seems to be associated with highest risk of miscarriage indicate that the jolts produced while exercising plays a role.

Inspite of the findings of this study, we do, however, think that it is too early to draw any public health inferences on this basis. Many positive effects of exercise are well established, and the findings of this study need to be replicated.

## Contribution to authorship

This study was initiated by A-M.N.A., and the analytical strategy was developed in collaboration with P.K.A., M.M., T.J., and M.L.J. M.M., T.J. and M.L.J. did all the data handling and statistical analyses. J.O.and M.J. contributed to methodological discussions, and P.K.A. addressed the statistical issues of the study.

M.M. drafted the first version of the paper and all of the co-authors revised the paper on several occasions.

## Details on ethics approval

The DNBC has been approved by the Danish Scientific Ethics Committee under ref. no (KF) 01-471/94, and this specific study has been approved by the Danish Data Protection Board.
